# Hospital Meetings, &c.

**Published:** 1897-07-31

**Authors:** 


### HOSPITAL MEETINGS, &C.

### PRIZE-GIVING TO THE STUDENTS OF THE LONDON SCHOOL OF DENTAL SURGERY.

On Tuesday, July 20th, the staff of the Dental Hospital, London, gave a most enjoyable and successful evening's entertainment at the Galleries of the Royal Society of British Artists on the occasion of the prize distribution by Sir Frank Lockwood, Q.C., M.P., to the students of the London School of Dental Surgery. The reception of the numerous guests took place at 8 p.m., and at 8.30 the Dean Smale commenced the business, or rather pleasure, of the evening, by a short, pithy speech, in which he touched on the increasing work of the hospital, the progress of the new building, the great need of money, and the help given by the staff and students of the hospital and other members of the profession. Sir Frank Lockwood then distributed the prizes. Mr. T. H. Miller, Mr. N. Miller, and Mr. Thew especially distinguished themselves; Mr. T. H. Miller, M.B., B.C.H., taking the Saunders' Scholarship and Ash's Prize, the first prize in dental anatomy, the first prize in dental surgery; while the first and second prizes in metallurgy were equally divided between him and Mr. T. W. Thew. In dental mechanics Mr. N. Miller took the first prize, and also first prize in operative dental surgery (Mr. T. W. Thew taking the second). The second prize in dental anatomy also fell to his share, his paper on this subject being worthy of a first prize had this not been carried off by Mr. T. H. Miller. Sir Frank Lockwood, speaking of the good work done by members of the hospital, congratulated them, pointing out that to ease pain in the man who works with either head or hand is to grant him more strength and vitality for his work, and that the gratuitous advice given to the poor by the hospital gave it great claim on the sympathy of the public. The School of Dentistry was in itself worthy of standing alone. Prevention is always better than cure, and the teeth of children should be periodically examined in order to avert after suffering. Sir Frank Lockwood went on to say that the prosperity of the hospital was proved by the fact that in the year 1874 19,255 cases were treated, and in 1896 the number of cases was 57,674. He wished them all future success in the good work. A hearty vote of thanks to Sir Frank Lockwood closed the formal proceedings.

